# Genotype and visceral adiposity interactions on hepatic steatosis in Mexican adults

**DOI:** 10.1210/jendso/bvag249

**Published:** 2026-10-27

**Authors:** Wendolyn E Dávila-Olmedo, Ana T Arias-Marroquín, Fabiola M Del Razo-Olvera, Paloma Almeda-Valdes, Ivette Cruz Bautista, Donaji V Gómez-Velasco, César E Lam-Chung, Alejandro Campos Muñóz, Berenice Rivera-Paredez, Paola Vázquez-Cárdenas, Gabriela A Galán-Ramírez, Alexandro J Martagón-Rosado, Liliana Juárez-Martínez, Rodrigo Otero-Otero, Francisco Álvarez-Molina, Alicia Huerta-Chagoya, Ana Ochoa-Guzmán, Teresa Tusie-Luna, Roopa Mehta, Ricardo U Macías-Rodriguez, Carlos A Aguilar-Salinas, Adrián Soto-Mota

**Affiliations:** Unidad de Investigación de Enfermedades Metabólicas, Instituto Nacional de Ciencias Médicas y Nutrición Salvador Zubirán, Mexico City 14080, Mexico; División de Estudios de Posgrado, Facultad de Medicina, Programa de Ciencias Médicas, Odontológicas y de la Salud, Universidad Nacional Autónoma de México (UNAM), Mexico City 04510, Mexico; Unidad de Investigación de Enfermedades Metabólicas, Instituto Nacional de Ciencias Médicas y Nutrición Salvador Zubirán, Mexico City 14080, Mexico; Tecnologico de Monterrey, Escuela de Medicina y Ciencias de la Salud, Mexico City 14380, Mexico; Unidad de Investigación de Enfermedades Metabólicas, Instituto Nacional de Ciencias Médicas y Nutrición Salvador Zubirán, Mexico City 14080, Mexico; División de Estudios de Posgrado, Facultad de Medicina, Programa de Ciencias Médicas, Odontológicas y de la Salud, Universidad Nacional Autónoma de México (UNAM), Mexico City 04510, Mexico; Department of Endocrinology and Lipid Metabolism, Instituto Nacional de Ciencias Médicas y Nutrición Salvador Zubirán, Mexico City 14080, Mexico; Unidad de Investigación de Enfermedades Metabólicas, Instituto Nacional de Ciencias Médicas y Nutrición Salvador Zubirán, Mexico City 14080, Mexico; Unidad de Investigación de Enfermedades Metabólicas, Instituto Nacional de Ciencias Médicas y Nutrición Salvador Zubirán, Mexico City 14080, Mexico; Unidad de Investigación de Enfermedades Metabólicas, Instituto Nacional de Ciencias Médicas y Nutrición Salvador Zubirán, Mexico City 14080, Mexico; División de Estudios de Posgrado, Facultad de Medicina, Programa de Ciencias Médicas, Odontológicas y de la Salud, Universidad Nacional Autónoma de México (UNAM), Mexico City 04510, Mexico; Unidad de Investigación de Enfermedades Metabólicas, Instituto Nacional de Ciencias Médicas y Nutrición Salvador Zubirán, Mexico City 14080, Mexico; Centro de Investigación en Políticas, Población y Salud, Facultad de Medicina, Universidad Nacional Autónoma de México (UNAM), Mexico City 04510, Mexico; Departamento de Biología Molecular e Histocompatibilidad, Subdirección de Investigación Biomédica, Hospital General Dr. Manuel Gea González, Mexico City 14080, Mexico; Unidad de Investigación de Enfermedades Metabólicas, Instituto Nacional de Ciencias Médicas y Nutrición Salvador Zubirán, Mexico City 14080, Mexico; Tecnologico de Monterrey, Escuela de Medicina y Ciencias de la Salud, Mexico City 14380, Mexico; Unidad de Investigación de Enfermedades Metabólicas, Instituto Nacional de Ciencias Médicas y Nutrición Salvador Zubirán, Mexico City 14080, Mexico; Tecnologico de Monterrey, Escuela de Medicina y Ciencias de la Salud, Mexico City 14380, Mexico; OriGen Health Research Center, University of Texas at Austin and Tecnologico de Monterrey, Austin, Texas 78712, USA; Unidad de Investigación de Enfermedades Metabólicas, Instituto Nacional de Ciencias Médicas y Nutrición Salvador Zubirán, Mexico City 14080, Mexico; Unidad de Investigación de Enfermedades Metabólicas, Instituto Nacional de Ciencias Médicas y Nutrición Salvador Zubirán, Mexico City 14080, Mexico; Unidad de Investigación de Enfermedades Metabólicas, Instituto Nacional de Ciencias Médicas y Nutrición Salvador Zubirán, Mexico City 14080, Mexico; Programs in Metabolism and Medical and Population Genetics, Broad Institute of Harvard and MIT, Cambridge, Massachusetts 2142, USA; Center for Genomic Medicine, Massachusetts General Hospital, Boston, Massachusetts 02114, USA; Diabetes Unit, Massachusetts General Hospital, Boston, MA 2114, USA; Unidad de Biología Molecular y Medicina Genómica, Instituto Nacional de Ciencias Médicas y Nutrición Salvador Zubirán, Mexico City 14080, Mexico; Unidad de Biología Molecular y Medicina Genómica, Instituto Nacional de Ciencias Médicas y Nutrición Salvador Zubirán, Mexico City 14080, Mexico; Departamento de Medicina Genómica y Toxicología Ambiental, Instituto de Investigaciones Biomédicas, UNAM, Mexico City 04510, Mexico; Department of Endocrinology and Lipid Metabolism, Instituto Nacional de Ciencias Médicas y Nutrición Salvador Zubirán, Mexico City 14080, Mexico; Division of Hepatology and Liver Transplant, Instituto Nacional de Ciencias Médicas y Nutrición Salvador Zubirán, Mexico City 14080, Mexico; Unidad de Investigación de Enfermedades Metabólicas, Instituto Nacional de Ciencias Médicas y Nutrición Salvador Zubirán, Mexico City 14080, Mexico; División de Estudios de Posgrado, Facultad de Medicina, Programa de Ciencias Médicas, Odontológicas y de la Salud, Universidad Nacional Autónoma de México (UNAM), Mexico City 04510, Mexico; Tecnologico de Monterrey, Escuela de Medicina y Ciencias de la Salud, Mexico City 14380, Mexico; Unidad de Investigación de Enfermedades Metabólicas, Instituto Nacional de Ciencias Médicas y Nutrición Salvador Zubirán, Mexico City 14080, Mexico; Tecnologico de Monterrey, Escuela de Medicina y Ciencias de la Salud, Mexico City 14380, Mexico

**Keywords:** *SLC16A11*, gene–environment interaction, MASLD, visceral adiposity, fatty liver index

## Abstract

**Background:**

The *SLC16A11* haplotype has been associated with type 2 diabetes (T2D) in populations with high Native American ancestry; however, its role in hepatic outcomes remains uncertain. We evaluated the association of rs13342232 with noninvasive liver markers and whether visceral adiposity tissue (VAT) modifies this relationship in Mexican adults with and without T2D.

**Methods:**

We included 607 adults (non-T2D, *n* = 256; T2D, *n* = 351) with genotype, VAT, and fatty liver index (FLI) data. Hepatic steatosis was additionally assessed using FibroScan-derived controlled attenuation parameter and steatosis grade in 301 participants. Multivariable regression models with VAT × rs13342232 interaction terms were fitted, adjusting for ancestry principal components.

**Results:**

A significant VAT × G/G interaction on FLI was observed in the full sample (*β* = 3.19, 95% CI 0.23-6.15; *P* = .035). The interaction was markedly stronger in adults without T2D (*β* = 7.51, 95% CI 1.23-13.78; *P* = .018) than in those with T2D (*β* = 2.00, 95% CI −0.43 to 4.42; *P* = .105), suggesting that the gene–environment effect is most clearly expressed before diabetes onset. Null findings for both the A/G interaction and the dominant model in the non-T2D stratum support a recessive inheritance pattern. FibroScan validation confirmed a VAT × G/G interaction on steatosis grade in non-T2D participants (*β* = .50, 95% CI 0.01-0.99; *P* = .044). In the T2D stratum, a significant negative A/G × VAT interaction was observed (*β* = −1.63, *P* = .046), indicating heterozygote attenuation of the VAT–steatosis relationship.

**Conclusion:**

The *SLC16A11* rs13342232 G/G genotype amplifies the hepatic steatosis response to visceral adiposity in Mexican adults. This effect is strongest prior to diabetes onset, supporting a gene–environment interaction in hepatic fat accumulation.

Genome-wide association studies have identified single-nucleotide variants (SNVs) linked to metabolic diseases, including obesity, type 2 diabetes (T2D), and liver disease (1). In 2014, the Slim Initiative in Genomic Medicine for the Americas Type 2 Diabetes Consortium identified the *SLC16A11* haplotype on chromosome 17p13.1 as a common risk factor for T2D in the Mexican population (2). The *SLC16A11* haplotype is characterized by 5 specific SNVs (rs117767867, rs13342692, rs13342232, rs75418188, and rs75493593) (2, 3). This risk haplotype is common among Hispanics/Latinos, particularly those with a higher proportion of Native American ancestry—estimated at around 50%—and is rare among individuals of European and African descent (3). *SLC16A11* mRNA is predominantly expressed in the liver, and its protein product is localized in the endoplasmic reticulum (2, 4). Suppression of *SLC16A11* expression in hepatocytes reduces its localization to the cell membrane, thus impairing its function and altering fatty acid metabolism, which results in increased intracellular levels of triacylglycerols (4).

The effect of the *SLC16A11* haplotype on lipid metabolism has been previously described. Almeda-Valdes et al explored its impact on the metabolic profiles of Mexican mestizo individuals with and without T2D. Their findings revealed that carriers of the risk haplotype who had T2D had significantly higher levels of γ-glutamyl transferase (GGT) and alanine aminotransferase (ALT) compared with noncarriers, after adjusting for gender, age, and ancestry (5). In addition, haplotype carriers exhibited lower fat-free mass (FFM) than noncarriers. Notably, among women with a normal body mass index (BMI), carriers also had larger adipocyte size (5). These results are consistent with data from a Mendelian randomization study showing that a greater visceral adipose tissue (VAT) mass is causally associated with a higher risk of metabolic dysfunction–associated steatotic liver disease (MASLD) (6). VAT was therefore selected as the exposure of interest in the present study, given that visceral adipocytes release free fatty acids preferentially into the portal circulation, directly increasing hepatic lipid influx and promoting insulin resistance, 2 central mechanism in MASLD pathogenesis. Moreover, VAT is a stronger predictor of hepatic fat content than subcutaneous adiposity, independent of BMI (7-9). This evidence supports a potential role for *SLC16A11* in hepatic lipid metabolism; however, its direct association with liver disease remains insufficiently explored, especially in individuals with T2D, where hepatic steatosis is prevalent.

Mexico has a genetically admixed population, shaped primarily by Amerindian, European, and, to a lesser extent, African ancestry (2, 10). This diversity poses a significant challenge in genetic association studies due to potential confounding from population stratification (11). Considering ancestry components is essential to disentangle the actual effects of genetic variants from those attributable to ancestral background (11, 12). This is particularly important for complex diseases such as MASLD (13). Although liver biopsy is the gold standard for the diagnosis and staging of MASLD, its invasive nature limits its use (14). Several noninvasive scores have been developed to estimate the presence of hepatic steatosis and stratify risk, permitting early identification of individuals at risk of progression to advanced fibrosis (15). Among them, the fatty liver index (FLI), which integrates simple anthropometric and biochemical parameters, has been widely validated as a surrogate marker of steatosis in clinical practice and epidemiological research (16). In this study, we evaluate whether the *SLC16A11* rs13342232 G/G genotype interacts with VAT to influence noninvasive markers of hepatic steatosis in Mexican adults with and without T2D. A validation subsample with noninvasive assessment of hepatic steatosis by transient elastography was used to replicate key findings.

## Materials and methods

### Study design and participants

This cross-sectional study was based on a secondary analysis of data derived from 2 independent observational studies conducted in adults aged ≥18 years residing in Central Mexico, who participated in research protocols at the Unidad de Investigación de Enfermedades Metabólicas (UIEM) of the Instituto Nacional de Ciencias Médicas y Nutrición Salvador Zubirán (INCMNSZ), in Mexico City. Participant recruitment was conducted between 2019 and 2025. All participants were born in Mexico and reported Mexican ancestry in both parents and grandparents.

One study enrolled adults with a diagnosis of T2D, whereas the other included adults without T2D. For the present analysis, participants from both studies were pooled and classified according to T2D status.

### Participants with T2D

Participants with T2D were selected from a well-characterized prospective cohort of Mexican adults originally established to evaluate cardiometabolic complications associated with the disease. Data from the baseline study visit were used for the present analysis. T2D diagnosis was self-reported. Exclusion criteria included other types of diabetes, known nondiabetic causes of kidney disease, hepatic failure, pregnancy, lactation, and a life expectancy of less than 1 year.

A total of 1167 individuals with T2D were invited to participate through external referrals and the diabetes clinic at the INCMNSZ. Of these, 12 were excluded due to a diagnosis other than T2D, 536 were excluded because genetic data were unavailable, and 185 were excluded due to incomplete anthropometric data. The final analytical sample comprised 351 adults with T2D. This study was approved by the Research Ethics Committee of the INCMNSZ (approval number: 2705). All research was conducted in accordance with the Declaration of Helsinki. All participants provided written informed consent.

### Individuals without T2D

Participants without T2D were recruited from a cross-sectional observational study of adults without a prior diagnosis of diabetes, conducted at the same institution (UIEM and INCMNSZ), designed to evaluate intrahepatic fat content according to the presence of the *SLC16A11* risk haplotype. Absence of T2D was confirmed biochemically through fasting plasma glucose and/or glycated hemoglobin (HbA1c) measured at the study visit, in addition to the absence of a self-reported prior diagnosis.

Exclusion criteria included a diagnosis of any type of diabetes, pregnancy or lactation, and unavailability of genetic data. This study was approved by the Research Ethics Committee of the INCMNSZ (approval number: 2944). The final analytical sample comprised 256 adults without T2D.

### Procedures

#### Clinical and biochemical data

Sociodemographic characteristics, medical history, and lifestyle factors were assessed using standardized questionnaires. Morning fasting venous blood samples (9-12 hours) were used to measure glycated hemoglobin (HbA1c), fasting glucose and insulin, lipid profile (including total cholesterol, low-density lipoprotein cholesterol, high-density lipoprotein cholesterol, and triglycerides), and hepatic enzymes, including ALT, aspartate aminotransferase, and GGT, as well as uric acid. All analyses were conducted in the Endocrinology Department at INCMNSZ using standardized protocols.

#### Anthropometric measures and body composition analysis

Anthropometric measurements were obtained using standardized protocols and calibrated equipment. Waist circumference was assessed at the midpoint between the last rib and the iliac crest using a 2 m Lufkin anthropometric tape. Body mass index was calculated as weight in kilograms divided by height in meters squared (kg/m^2^). Body composition was assessed using a bioelectrical impedance analyzer (SECA mBCA 514, Hamburg, Germany). Body weight and height were measured using the integrated scale and stadiometer of the same device. All scans were performed and interpreted by certified technicians. Estimates of total fat mass (%) and FFM (kg) were obtained, along with VAT volume, reported in liters (L).

#### Hepatic steatosis assessment and MASLD classification

Hepatic steatosis was assessed using 2 complementary approaches. As the primary noninvasive surrogate marker, the FLI was calculated according to the formula proposed by Bedogni et al: FLI = (*e^L^*/(1 + *e^L^*)) × 100, where *L* = 0.953 × ln[triglycerides (mg/dL)] + 0.139 × BMI (kg/m^2^) + 0.718 × ln[GGT (U/L)] + 0.053 × waist circumference (cm) − 15.745 (13). An FLI ≥ 60 was used as indicative of significant hepatic fat accumulation. In a subset of 301 participants with available transient elastography data, hepatic steatosis was additionally quantified using the controlled attenuation parameter (CAP; dB/m) and steatosis grade (S0-S3), with significant steatosis defined as CAP ≥ 268 dB/m (≥S1), obtained by FibroScan (17).

Metabolic dysfunction–associated steatotic liver disease was defined according to the 2023 multi-society international nomenclature consensus. Participants were classified as having MASLD if they met 2 conditions simultaneously: (1) evidence of hepatic steatosis by at least one of the above markers (FLI ≥ 60 or CAP ≥ 268 dB/m) and (2) the presence of at least one cardiometabolic risk factor, defined as T2D, overweight or obesity (BMI ≥ 25 kg/m^2^), arterial hypertension, dyslipidemia, or other features of metabolic syndrome (13). Participants with hepatic steatosis but without any cardiometabolic risk factor were not classified as MASLD and were excluded from steatosis-related outcome analyses.

### Genotyping and ancestry data

Genomic data were obtained from the Slim Initiative in Genomic Medicine for the Americas Type 2 Diabetes Consortium, as previously described (2). Briefly, genomic DNA was extracted from whole blood using the QIAamp 96 DNA Blood Kit (Qiagen), and genotyping was performed at the Broad Institute Genetic Analysis Platform using the Illumina HumanOmni2.5-4v1_B SNP array. Only genotype data for the 5 *SLC16A11* haplotype SNVs and the principal components (PCs) derived from genome-wide genotype data were made available to the research team under authorized access granted by UIEM and INCMNSZ. These PCs were used in the present analysis to estimate ancestry and correct for population stratification. Genotypic data were kept blinded to researchers involved in participant recruitment and study procedures until completion of data collection, after which they were merged with the newly collected measurements for statistical analysis. Participants lacking valid genotype data were excluded from the analytical sample, accounting for the substantial proportion excluded on this basis.

### Statistical analysis

All statistical analyses were performed in R (version 4.5.2). Continuous variables are reported as median (interquartile range) given non-normal distributions. Categorical variables are reported as frequencies and percentages. Between-group comparisons were performed using the Mann–Whitney *U* test for continuous variables and the χ^2^ test for categorical variables when comparing 2 groups (T2D vs non-T2D); Kruskal–Wallis tests were used for comparisons across 3 genotype groups (A/A, A/G, and G/G). Conformity of the rs13342232 genotype distribution with Hardy–Weinberg equilibrium was assessed using an exact test, implemented via the HardyWeinberg R package, both in the overall analytical sample and stratified by T2D status.

Missing data in anthropometric predictors (weight, height, waist circumference, VAT, fat mass, and FFM) were imputed using random forest-based multiple imputation via the missForest package (set.seed = 123), which simultaneously handles continuous and categorical variables without distributional assumptions. Out-of-bag normalized root mean squared error and proportion of falsely classified entries were monitored across 5 iterations to assess convergence (18). Laboratory values, genetic variables, and outcome variables were not imputed.

Haplotype-wide analyses were conducted using the imputed dataset (*n* = 619), whereas the primary analyses were restricted to participants with complete genotype, VAT and FLI data (*n* = 607).

To examine the study hypothesis (that the *SLC16A11* rs13342232 genotype moderates the effect of VAT on hepatic fat accumulation), we fitted a series of multivariable linear regression models with FLI as the primary outcome. The main interaction model included VAT, rs13342232 genotype (A/A as reference), their 2-way interaction terms (VAT × A/G and VAT × G/G), and the following covariates: Native American ancestry proportion (%), age (years), sex, BMI (kg/m^2^), and T2D status (in the full-sample model). Stratified analyses by T2D status were performed to evaluate whether the VAT × genotype interaction differed by metabolic context. A dominant genetic model (A/A vs A/G + G/G) was additionally fitted as a sensitivity analysis.

FibroScan-based outcomes (CAP and steatosis grade) were analyzed in the 301-participant subsample using analogous linear regression models. A 3-way interaction model (VAT × rs13342232 × T2D) was additionally fitted in the full sample to formally evaluate effect modification by diabetes status. Marginal predicted values and interaction plots were generated using the ggeffects package. All figures were produced using ggplot2 and assembled with patchwork.

To assess potential multicollinearity, generalized variance inflation factors (GVIFs) were computed for all interaction models using the car package. For models containing categorical predictors and interaction terms, the adjusted GVIF^(1/(2 × Df)) was used as the directly comparable index, with values below 3.16 (ie, √10) considered acceptable. All covariates, including BMI, VAT, and the ancestry PCs, returned adjusted GVIF values well within this threshold (range 1.04-2.07 for covariates), confirming that the inclusion of BMI as both a covariate and a component of FLI did not introduce problematic collinearity. Interaction terms showed adjusted GVIF values between 2.66 and 3.13, also below the threshold, indicating that the SNP × VAT product terms did not distort estimation of other model coefficients. As an additional specificity control, a permutation-based dummy gene analysis was performed: the genotype labels of rs13342232 were randomly reassigned across participants (preserving observed allele frequencies) and the full interaction model was refitted. The dummy SNP showed no significant interaction with VAT across any outcome (all interaction term *P* > .20), confirming that the significant SNP × VAT interaction effects observed in the real models reflect a genotype-specific biological relationship rather than an artifact of the model structure.

### Genetic ancestry estimation

To account for population stratification and characterize the genetic ancestry composition of our study sample, we estimated individual-level European and Native American ancestry proportions from PCs derived from genome-wide genotype data. Principal component analysis had been previously performed on quality-controlled genotype data, yielding PC1 and PC2 values for ancestry (PC1Ancestría, PC2Ancestría). Consistent with established findings in Mexican populations (1), PC1 primarily discriminates between European and Native American ancestral components, with more negative values indicating higher Native American ancestry and more positive values indicating higher European ancestry.

To convert PC1 values to ancestry proportions, we applied a linear projection method calibrated to expected Mexican mestizo ancestry ranges. PC1 values were first normalized to a 0-1 scale using min-max normalization. We then applied a linear transformation mapping normalized PC1 values to ancestry proportions, with the transformation calibrated to produce ancestry estimates within the range of 35% to 75% Native American ancestry, consistent with published Mexican population data (2, 3). The transformation equation was

Native American % = 75% − (PC1_normalized × 40%)

European % = 100% − Native American %

This calibration was based on published estimates showing that Mexican mestizos exhibit ∼50% to 65% Native American and 35% to 45% European ancestry, with regional variation (4, 5). African ancestry, which typically comprises 2% to 6% of Mexican genetic ancestry, was not estimated in this analysis. The ancestry estimates were validated by comparison with published Mexican ancestry proportions. Our sample showed a mean Native American ancestry of 56.5% (SD = 7.8%, range: 35-75%), consistent with expected values for Mexican populations from the central and southern regions of Mexico (4, 5).

For analyses examining associations with ancestry, individuals were categorized into 4 groups based on Native American ancestry proportion: Higher European (<45%), balanced (45-55%), higher native (55-65%), and predominantly native (>65%). This PC-based approach provides approximate ancestry estimates suitable for population stratification correction and descriptive analyses.

## Results

### Participants’ characteristics by T2D status and genotype

Participants with T2D were significantly older (median 60 vs 45 years, *P* < .001), had higher VAT (3.1 vs 2.7 L, *P* < .001), higher FLI (61.4 vs 54.9, *P* < .001), higher HbA1c (8.2% vs 5.7%, *P* < .001), and a greater prevalence of hepatic steatosis by FLI ≥ 60 (51.6% vs 44.5%, *P* = .103; Table 1). Among FibroScan-assessed participants, T2D was associated with higher CAP (296 vs 265 dB/m, *P* = .002) and greater liver stiffness (4.7 vs 4.3 kPa, *P* = .007).

**Table 1:** Characteristics of participants stratified by T2D status (*n* = 607)

	Overall (*n* = 607)	No T2D (*n* = 256)	T2D (*n* = 351)	*P*-value
Anthropometrics				
Age, years	54.0 (43.0-62.5)	45.0 (31.0-53.0)	60.0 (52.0-67.0)	**<**.**001**
BMI, kg/m^2^	28.1 (25.6-31.8)	27.9 (25.3-31.3)	28.3 (25.8-31.9)	.138
Waist circumference, cm	94.5 (86.5-103.2)	92.0 (82.0-99.7)	95.5 (90.0-105.5)	**<**.**001**
VAT, L	2.9 (2.1-3.9)	2.7 (1.9-3.6)	3.1 (2.3-4.2)	**<**.**001**
Fat mass, %	38.3 (31.9-43.8)	37.5 (30.3-42.8)	38.7 (32.3-44.5)	.**015**
Fat-free mass, kg	43.9 (38.3-52.6)	44.6 (39.0-54.4)	43.6 (37.4-51.1)	.**008**
Metabolic				
Glucose, mg/dL	109.0 (95.0-153.0)	96.5 (90.0-105.0)	140.0 (109.0-191.0)	**<**.**001**
Insulin, µU/mL	10.8 (6.7-17.4)	9.2 (5.9-13.8)	12.3 (7.7-19.7)	**<**.**001**
HbA1c, %	6.4 (5.7-8.6)	5.7 (5.4-5.9)	8.2 (6.8-10.1)	**<**.**001**
Triglycerides, mg/dL	156.0 (107.0-219.5)	132.5 (87.8-187.0)	164.0 (130.0-237.5)	**<**.**001**
ALT, U/L	24.0 (18.0-32.5)	25.0 (18.0-34.0)	23.0 (18.0-32.0)	.277
AST, U/L	24.0 (20.0-29.0)	24.0 (21.0-30.0)	23.0 (19.0-28.0)	.008
GGT, U/L	21.0 (16.0-32.0)	21.0 (15.0-32.0)	21.0 (16.0-32.0)	.838
ALT/AST ratio	1.00 (0.84-1.25)	1.00 (0.84-1.22)	1.00 (0.84-1.26)	.589
FLI	58.3 (32.0-82.1)	54.9 (24.5-76.1)	61.4 (38.1-84.0)	**<**.**001**
FibroScan (*n* = 301)				
CAP, dB/m	276.0 (225.0-311.0)	265.0 (217.0-306.5)	296.0 (266.0-336.0)	.**002**
Steatosis grade	1.0 (0.0-3.0)	2.0 (0.0-3.0)	1.0 (0.0-3.0)	.274
Liver stiffness, kPa	4.3 (3.6-5.3)	4.3 (3.5-5.1)	4.7 (4.0-6.0)	.**007**
Clinical, *n* (%)				
Male sex	219 (36.1)	71 (27.7)	148 (42.2)	**<**.**001**
MASLD (FLI ≥60)	295 (48.6)	114 (44.5)	181 (51.6)	.103
Obesity (BMI ≥30)	224 (36.9)	90 (35.2)	134 (38.2)	.499
*SLC16A11* rs13342232 genotype, *n* (%)				
A/A (protective)	249 (41.0)	114 (44.5)	135 (38.5)	NA
A/G (heterozygous)	265 (43.7)	116 (45.3)	149 (42.5)	NA
G/G (risk)	93 (15.3)	26 (10.2)	67 (19.1)	NA

Data are presented as median (interquartile range) for continuous variables and *n* (%) for categorical variables. *P*-values compare participants with and without T2D and were calculated using the Mann–Whitney *U* test for continuous variables and the χ^2^ test for categorical variables. FibroScan measurements were available in 301 participants (no T2D, *n* = 222; T2D, *n* = 79). Statistically significant *P* values (*P* < .05) are shown in bold.

Abbreviations: ALT, alanine aminotransferase; AST, aspartate aminotransferase; BMI, body mass index; CAP, controlled attenuation parameter; FLI, fatty liver index; GGT, γ-glutamyl transferase; HbA1c, glycated hemoglobin; MASLD, metabolic dysfunction–associated steatotic liver disease; NA, not applicable; T2D, type 2 diabetes; VAT, visceral adipose tissue.

Stratified by genotype, G/G homozygotes showed significantly higher T2D prevalence (72.0% vs 54.2% in A/A; *P* = .009) and HbA1c (median 7.3% vs 6.2%; *P* = .017), consistent with the established diabetogenic effect of the risk allele (Table 2). No significant between-genotype differences were observed in anthropometric measures, liver enzymes, VAT, or FLI in unadjusted comparisons (all *P* > .05). The Venn diagram revealed that 26.4% of the analytical samples had T2D alone, 9.4% had overlapping hepatic steatosis and T2D, and 20.4% had concurrent hepatic steatosis, T2D, and obesity (Fig. S1) (19), illustrating the substantial metabolic co-morbidity burden in this population.

**Table 2:** Characteristics of participants stratified by *SLC16A11* rs13342232 genotype (*n* = 607)

	Overall (*n* = 607)	A/A (*n* = 249)	A/G (*n* = 265)	G/G (*n* = 93)	*P*-value
Anthropometrics					
Age, years	54.0 (43.0-62.5)	54.0 (44.0-64.0)	54.0 (42.0-61.0)	57.0 (45.0-64.0)	.221
BMI, kg/m^2^	28.1 (25.6-31.8)	27.9 (25.2-31.8)	28.4 (25.7-31.9)	28.0 (26.3-31.2)	.747
Waist circumference, cm	94.5 (86.5-103.2)	93.7 (87.5-103.0)	95.0 (84.8-103.5)	94.0 (88.0-103.3)	.97
VAT, L	2.9 (2.1-3.9)	3.0 (2.2-3.9)	2.8 (2.1-4.1)	2.8 (2.2-3.8)	.852
Fat mass, %	38.3 (31.9-43.8)	38.5 (32.0-43.7)	38.1 (31.6-44.0)	38.3 (31.9-42.7)	.987
Fat-free mass, kg	43.9 (38.3-52.6)	44.0 (38.7-51.1)	44.0 (38.1-53.9)	42.8 (37.4-50.2)	.232
Metabolic					
Glucose, mg/dL	109.0 (95.0-153.0)	107.0 (96.0-150.0)	109.0 (94.0-148.0)	115.0 (97.0-176.0)	.175
Insulin, µU/mL	10.8 (6.7-17.4)	10.3 (6.8-16.2)	10.7 (6.5-17.4)	12.0 (6.7-20.6)	.192
HbA1c, %	6.4 (5.7-8.6)	6.2 (5.7-8.2)	6.2 (5.6-8.7)	7.3 (5.9-9.0)	.**017**
Triglycerides, mg/dL	156.0 (107.0-219.5)	161.0 (103.0-231.0)	155.0 (109.0-216.0)	145.0 (108.0-202.0)	.615
ALT, U/L	24.0 (18.0-32.5)	23.0 (18.0-32.0)	24.0 (17.0-32.0)	24.0 (19.0-33.0)	.596
AST, U/L	24.0 (20.0-29.0)	23.0 (20.0-28.0)	24.0 (20.0-29.0)	24.0 (21.0-29.0)	.675
GGT, U/L	21.0 (16.0-32.0)	22.0 (16.0-32.0)	20.0 (15.0-31.0)	22.0 (16.0-31.0)	.693
ALT/AST ratio	1.00 (0.84-1.25)	1.00 (0.84-1.21)	1.00 (0.84-1.26)	1.06 (0.88-1.27)	.435
FLI	58.3 (32.0-82.1)	58.3 (33.6-82.2)	62.6 (31.8-82.3)	52.8 (32.0-79.3)	.828
FibroScan (*n* = 301)					
CAP, dB/m	276.0 (225.0-311.0)	276.5 (224.0-307.3)	265.0 (228.5-313.0)	289.0 (227.0-312.0)	.846
Steatosis grade	1.0 (0.0-3.0)	1.5 (0.0-3.0)	1.0 (0.0-3.0)	1.0 (0.0-3.0)	.926
Liver stiffness, kPa	4.3 (3.6-5.3)	4.4 (3.6-5.4)	4.3 (3.5-5.2)	4.4 (3.9-5.3)	.64
Clinical, *n* (%)					
Male sex	219 (36.1)	87 (34.9)	92 (34.7)	40 (43.0)	.318
T2D	351 (57.8)	135 (54.2)	149 (56.2)	67 (72.0)	.**009**
MASLD (FLI ≥60)	295 (48.6)	118 (47.4)	137 (51.7)	40 (43.0)	.312
Obesity (BMI ≥30)	224 (36.9)	95 (38.2)	99 (37.4)	30 (32.3)	.591

Data are presented as median (interquartile range) for continuous variables and *n* (%) for categorical variables. Between-group comparisons were performed using the Kruskal–Wallis test for continuous variables and χ^2^ test for categorical variables. FibroScan data are available in 301 participants. Statistically significant *P* value (*P* < .05) are shown in bold.

Abbreviations: ALT, alanine aminotransferase; AST, aspartate aminotransferase; BMI, body mass index; CAP, controlled attenuation parameter; FLI, fatty liver index; GGT, γ-glutamyl transferase; MASLD, metabolic dysfunction–associated steatotic liver disease; T2D, type 2 diabetes; VAT, visceral adipose tissue.

### VAT × rs13342232 interaction on FLI

The central hypothesis (that the effect of VAT on hepatic fat accumulation is modulated by the *SLC16A11* genotype) was supported across all model specifications (Table 3, Fig. 1). In the full analytical sample (*n* = 607), a significant positive interaction between VAT and G/G genotype was observed (*β* = 3.192, 95% CI 0.231 to 6.153; *P* = .035), indicating that each additional liter of VAT was associated with a 3.19 point greater increase in FLI among G/G homozygotes compared with A/A wild-type carriers.

**Figure 1:**
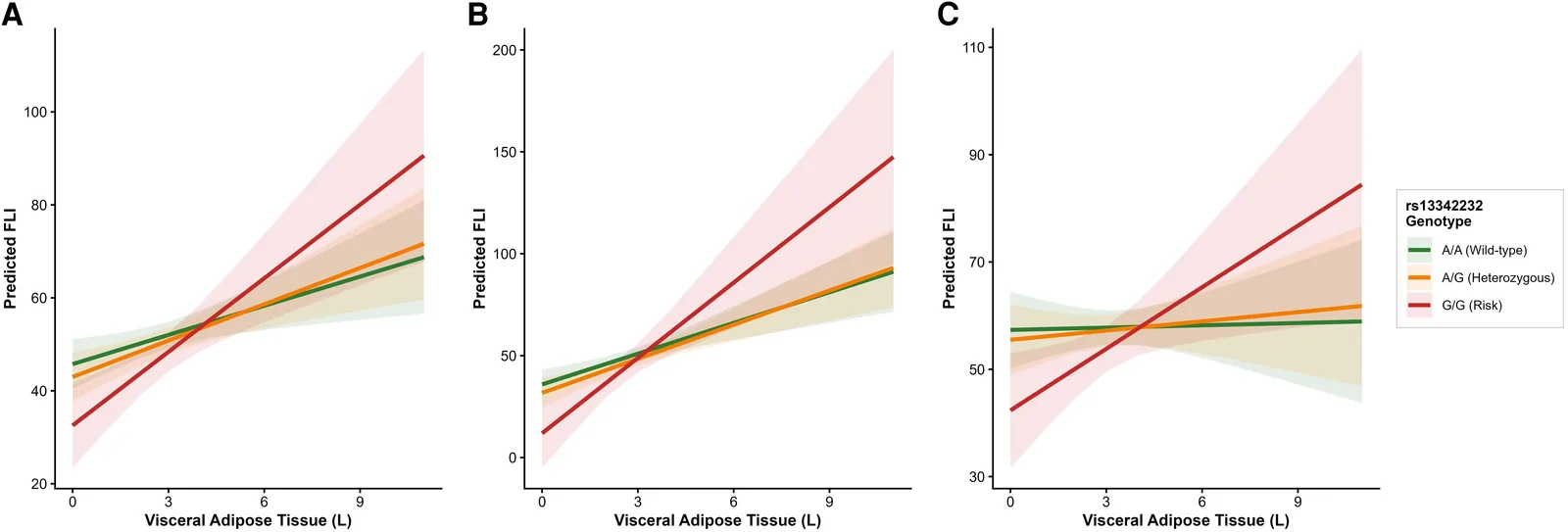
Interaction between visceral adipose tissue (VAT) × rs13342232 genotype on the fatty liver index (FLI). Marginal predicted values of the FLI according to VAT (liters) are shown for each rs13342232 genotype (A/A, wild type; A/G, heterozygous; G/G, risk homozygous). (A) Full analytic sample (*n* = 607). (B) Participants without type 2 diabetes (T2D; *n* = 256). (C) Participants with T2D (*n* = 351). Predicted values were estimated from multivariable linear regression models adjusted for Native American ancestry (%), age, sex, and body mass index (BMI); the full-sample model additionally adjusted for T2D status. Shaded areas represent 95% CIs. The A/A genotype was used as the reference category.

**Table 3:** VAT × *SLC16A11* rs13342232 interaction on FLI: stratified by T2D status (*n* = 607)

	M4-full (*n* = 607)	M4–no T2D (*n* = 256)	M4-T2D (*n* = 351)
	*β* (95% CI)	*β* (95% CI)	*β* (95% CI)
Main effects			
VAT, L	2.091 (0.605, 3.578)**	5.008 (2.67, 7.35)***	.184 (−1.740, 2.108)
A/G (ref.: A/A)	−2.778 (−9.162, 3.606)	.567 (−1.32, 2.82)	−1.756 (−10.910, 7.399)
G/G (ref.: A/A)	−13.218 (−23.113, −3.323)**	−23.545 (−40.894, −6.196)**	−14.927 (−27.491, −2.362)*
VAT × genotype interaction			
VAT × A/G	.520 (−1.260, 2.299)	.467 (−2.074, 3.007)	−1.634 (−3.24, −0.03)*
VAT × G/G	3.192 (0.231, 6.153)*	7.506 (1.23, 13.78)*	1.996 (−0.43, 4.42)
Covariates			
Native American ancestry, %	.271 (0.019, 0.522)*	2.148 (0.703, 3.594)**	.171 (−0.088, 0.430)
Age, years	.063 (−0.040, 0.166)	.056 (−0.088, 0.200)	−0.036 (−0.184, 0.111)
Sex (male)	7.511 (4.561, 10.461)***	6.816 (1.920, 11.712)**	7.462 (3.860, 11.065)***
BMI, kg/m^2^	4.133 (3.823, 4.443)***	3.782 (3.247, 4.318)***	4.130 (3.755, 4.504)***
T2D	3.770 (0.700, 6.840)*	—	—
Model fit			
*R*^2^	.704	.752	.679
Adjusted *R*^2^	.699	.743	.67
*n*	607	256	351
*F*-statistic (*P*-value)	141.5 (<.001)	82.96 (<.001)	80.01 (<.001)

Regression coefficients (*β*) represent unstandardized regression estimates with 95% CI (*β* ± 1.96 × SE). Reference category for rs13342232 is A/A. Interaction terms represent the additional effect of VAT on FLI according to genotype relative to the A/A genotype. “M4-Full” included all participants; “M4–No T2D” included participants without T2D; and “M4-T2D” included participants with T2D. Models were adjusted for Native American ancestry, age, sex, BMI, and T2D status, as appropriate. Native American ancestry (%) was retained as the sole ancestry covariate. The T2D covariate was included only in the full model.

Abbreviations: BMI, body mass index; FLI, fatty liver index; T2D, type 2 diabetes; VAT, visceral adipose tissue.

*P < .05, **P < .01, ***P < .001.

The main effect of VAT on FLI was *β* = 2.091 (95% CI 0.605-3.578; *P* = .006) in A/A carriers. In contrast, G/G homozygotes showed a statistically significant negative effect on FLI (*β* = −13.218; 95% CI −23.113 to −3.323; *P* < .01), whereas no significant interaction was observed for A/G heterozygotes (*β* = −2.778, 95% CI −9.162 to 3.606; Table 3, Fig. 1).

Stratified analyses by T2D status revealed markedly different patterns (Table 3). In participants without T2D (*n* = 256), the VAT × G/G interaction was particularly pronounced (*β* = 7.506, 95% CI 1.23-13.78; *P* = .018), and VAT itself was a strong independent predictor of FLI (*β* = 5.008, 95% CI 2.67-7.35; *P* < .001). No significant interaction was observed for A/G heterozygotes in this stratum (*β* = .567, *P* = .665), and the dominant model (A/A vs A/G + G/G) yielded a null interaction (*P* = .372), consistent with a recessive inheritance pattern in which 2 copies of the G allele are required to amplify the VAT–steatosis relationship. In T2D participants (*n* = 351), the G/G × VAT interaction was attenuated and did not reach statistical significance (*β* = 1.996, 95% CI −0.43 to 4.42; *P* = .105). Notably, a significant negative A/G × VAT interaction was observed in this stratum (*β* = −1.634, 95% CI −3.24 to −0.03; *P* = .046), indicating that in the diabetic context, heterozygotes show an attenuated VAT–FLI relationship relative to A/A carriers. Importantly, G/G genotype was associated with significantly lower FLI in both strata (non-T2D: *β* = −23.545, *P* = .008; T2D: *β* = −14.927, *P* = .020), indicating lower baseline hepatic fat accumulation at low VAT values that becomes markedly amplified as visceral adiposity increases, particularly in the absence of diabetes.

### FibroScan validation: VAT × rs13342232 on steatosis measures

Characteristics of the FibroScan subsample did not differ significantly across genotype groups (all *P* > .05; Table 4), except for trends toward higher fasting glucose (*P* = .079) and HbA1c (*P* = .085) in G/G carriers, replicating the pattern observed in the full analytical sample. Type 2 diabetes was associated with lower steatosis grade in FibroScan models (*β* = −.611, *P* = .009; Table 5). In the non-T2D subsample specifically (Table 6), the VAT × G/G interaction on FibroScan steatosis grade was statistically significant (*β* = .502, 95% CI 0.01-0.99; *P* = .044), confirming the FLI-based finding using a biologically independent instrument. The interaction on CAP trended in the same direction (G/G × VAT: *β* = 18.89, *P* = .097) but did not reach statistical significance, consistent with CAP's lower sensitivity for detecting mild steatosis compared with grade-based classification. No significant G/G × VAT interaction was observed for liver stiffness (kPa) in either stratum (all *P* > .19), confirming that this locus influences lipid accumulation but not the fibrotic cascade (Table 6).

**Table 4:** Characteristics of FibroScan subsample by *SLC16A11* rs13342232 genotype (*n* = 301)

	Overall (*n* = 301)	A/A (*n* = 136)	A/G (*n* = 131)	G/G (*n* = 34)	*P*-value
Anthropometrics					
Age, years	48.0 (35.0-55.0]	49.5 (35.8-56.0)	45.0 (32.0-54.0)	46.0 (39.3-55.8)	0.198
BMI, kg/m^2^	27.9 (25.4-31.5)	27.9 (25.1-31.3)	28.5 (25.5-31.7)	27.1 (26.3-29.4)	.763
Waist circumference, cm	92.2 (83.5-101.0)	92.0 (84.3-100.2)	93.2 (82.0-102.3)	91.3 (83.1-98.2)	.704
VAT, L	2.8 (2.0-3.8)	2.9 (2.1-3.8)	2.7 (1.9-4.1)	2.7 (1.9-3.6)	.605
Fat mass, %	37.5 (31.1-42.9)	37.7 (31.0-42.3)	37.9 (31.3-43.5)	35.4 (30.6-43.1)	.732
Fat-free mass, kg	44.4 (38.8-55.0)	45.9 (39.5-53.8)	43.6 (38.7-56.7)	42.9 (38.2-51.3)	.445
Metabolic					
Glucose, mg/dL	98.0 (91.0-109.0)	99.0 (92.0-108.3)	96.0 (90.0-107.5)	101.0 (94.0-116.5)	.079
HbA1c, %	5.7 (5.4-6.1)	5.8 (5.5-6.1)	5.7 (5.3-6.1)	5.9 (5.6-6.4)	.085
Triglycerides, mg/dL	136.0 (92.0-188.0)	142.5 (88.5-200.0)	136.0 (94.0-187.0)	132.0 (89.8-181.3)	.817
ALT, U/L	24.0 (18.0-33.0)	23.0 (17.8-31.3)	26.0 (18.0-36.0)	24.0 (19.3-32.5)	.331
AST, U/L	24.0 (21.0-29.0)	23.0 (20.0-28.0)	24.0 (20.5-31.0)	25.0 (22.0-28.0)	.41
GGT, U/L	21.0 (15.0-31.0)	21.0 (16.0-30.0)	20.0 (15.0-32.5)	19.0 (14.3-28.5)	.706
ALT/AST ratio	1.00 (0.84-1.25)	1.00 (0.83-1.20)	1.04 (0.85-1.36)	1.04 (0.87-1.14)	.46
FLI	54.4 (27.0-79.3)	56.4 (29.6-75.2)	56.4 (24.4-80.6)	43.4 (28.6-74.7)	.688
FibroScan					
CAP, dB/m	276.0 (225.0-311.0)	276.5 (224.0-307.3)	265.0 (228.5-313.0)	289.0 (227.0-312.0)	.846
Liver stiffness, kPa	4.3 (3.6-5.3)	4.4 (3.6-5.4)	4.3 (3.5-5.2)	4.4 (3.9-5.3)	.64
Steatosis grade	1.0 (0.0-3.0)	1.5 (0.0-3.0)	1.0 (0.0-3.0)	1.0 (0.0-3.0)	.926
Clinical, *n* (%)					
Male sex	91 (30.2)	39 (28.7)	40 (30.5)	12 (35.3)	.75
MASLD (FLI ≥60)	134 (44.5)	59 (43.4)	64 (48.9)	11 (32.4)	.212
Obesity (BMI ≥30)	104 (34.6)	49 (36.0)	48 (36.6)	7 (20.6)	.191
T2D	45 (15.0)	22 (16.2)	15 (11.5)	8 (23.5)	.184

Data are presented as median (interquartile range) for continuous variables and *n* (%) for categorical variables. Between-group comparisons were performed using the Kruskal–Wallis test for continuous variables and χ^2^ test for categorical variables. Only participants with available FibroScan measurements were included in this analysis (*n* = 301). Glucose (*P* = .079) and HbA1c (*P* = .085) showed trends toward higher values in G/G, consistent with the main analytic sample.

Abbreviations: ALT, alanine aminotransferase; AST, aspartate aminotransferase; BMI, body mass index; CAP, controlled attenuation parameter; FLI, fatty liver index; GGT, γ-glutamyl transferase; MASLD, metabolic dysfunction–associated steatotic liver disease; T2D, type 2 diabetes; VAT, visceral adipose tissue.

*P < .05, **P < .01, ***P < .001.

**Table 5:** VAT × *SLC16A11* rs13342232 interaction on CAP, steatosis grade, and FLI in the FibroScan subsample

	Model-CAP (*n* = 301)	Model-steatosis (*n* = 301)	Model-FLI (*n* = 301)
	*β* (95% CI)	*β* (95% CI)	*β* (95% CI)
Main effects			
VAT, L	16.650 (9.807, 23.493)***	.244 (0.095, 0.392)**	4.479 (2.330, 6.627)***
A/G (ref.: A/A)	7.176 (−23.882, 38.234)	−.317 (−0.989, 0.356)	−5.227 (−13.397, 2.944)
G/G (ref.: A/A)	−23.281 (−83.938, 37.376)	−1.316 (−2.632, −0.000)****	−15.347 (−31.299, 0.606)****
VAT × genotype interaction			
VAT × A/G	−.120 (−9.034, 8.794)	.098 (−0.096, 0.292)	1.069 (−1.277, 3.415)
VAT × G/G	11.292 (−9.279, 31.863)	.526 (0.080, 0.972)*	4.654 (−0.756, 10.064)****
Covariates			
Age, years	.520 (0.010, 1.030)*	.012 (0.001, 0.023)*	.033 (−0.101, 0.167)
Sex (male)	−13.507 (−29.875, 2.861)	−.240 (−0.595, 0.115)	6.420 (1.825, 11.016)**
BMI, kg/m^2^	—	—	3.842 (3.352, 4.330)***
T2D	12.757 (−8.190, 33.705)	−.611 (−1.066, −0.156)**	−.693 (−6.202, 4.815)
Native American ancestry, *n* (%)	2.437 (0.212, 4.662)*	.047 (−0.001, 0.095)****	.433 (−0.153, 1.018)
Model fit			
*R*^2^	.208	.169	.741
Adjusted *R*^2^	.184	.143	.732
*n*	301	301	301
*F*-statistic (*P*-value)	8.51 (<.001)	6.57 (<.001)	82.77 (<.001)

Regression coefficients (*β*) represent unstandardized estimates with 95% CI (*β* ± 1.96 × SE). Models included an interaction term between visceral adipose tissue (VAT) and the *SLC16A11* rs13342232 genotype. The reference genotype was A/A. Analyses were performed in participants with available FibroScan measurements (*n* = 301). Models evaluating controlled attenuation parameter (CAP) and steatosis grade included only participants with available CAP and steatosis grade data (no T2D, *n* = 222; T2D, *n* = 79). The dash (—) indicates that the variable was not included in the model.

Abbreviations: BMI, body mass index; FLI, fatty liver index; T2D, type 2 diabetes.

*P < .05, **P < .01, ***P < .001, ****P < .10 (trend).

**Table 6:** Summary of VAT × *SLC16A11* genotype interaction analyses across the primary and validation cohorts

Interaction	Outcome	*Β*	95% CI	*P*-value	Sample
FibroScan validation—VAT × rs13342232 (G/G genotype)					
G/G × VAT	Steatosis grade	.502	0.01-0.99	.044	Non-T2D subsample
G/G × VAT	CAP (dB/m)	18.89	—	.097	Non-T2D subsample
G/G × VAT	Liver stiffness (kPa)	—	—	>.19	Both strata
Haplotype-wide SNP analyses—FLI (full cohort, *n* = 619 imputed)					
T/T (homozygote) × VAT	FLI	+4.07 to +4.24	—	≤.002	rs75493593, rs75418188, rs117767867
T/G (heterozygote) × VAT	FLI	−1.90 to −1.94	—	≈.016	rs75493593, rs75418188, rs117767867

Regression coefficients (*β*) represent interaction estimates. Models were adjusted for principal components (PC1-PC10), age, sex, and body mass index (BMI). Dashes (—) indicate values not reported or not applicable.

Abbreviations: CAP, controlled attenuation parameter; FLI, fatty liver index; T2D, type 2 diabetes; VAT, visceral adipose tissue.

### Haplotype-wide SNP analyses and linkage disequilibrium pattern

Interaction models were fitted for all 5 *SLC16A11* haplotype variants in the full cohort (*n* = 619, imputed dataset). Three SNPs—rs75493593, rs75418188, and rs117767867—produced virtually identical interaction estimates and *P*-values across all outcomes, consistent with high linkage disequilibrium among them. For FLI, the T/T (risk-homozygote) × VAT interaction was strongly significant for all 3 (*β* ≈ +4.07 to +4.24; *P* ≤ .002, Table 6), indicating that T/T homozygotes show markedly amplified VAT–steatosis coupling. The T/G heterozygote × VAT interaction was negative and significant for all 3 (*β* ≈ −1.90 to −1.94; *P* ≈ .016), suggesting a nonadditive (overdominant) pattern in the T2D-enriched cohort. For rs13342692, the C/T × VAT interaction on FLI was significant (*β* = −3.630, *P* = .001), and the direction mirrored that of rs13342232 with reversed allele coding, consistent with rs13342692 and rs13342232 representing complementary strand annotations of the same or closely proximate locus.

### Study population and genetic ancestry

A total of 607 participants with complete genotype, VAT, and FLI data constituted the analytical sample (non-T2D, *n* = 256; T2D, *n* = 351). A FibroScan subsample was available for 301 participants (no T2D, *n* = 222; T2D, *n* = 79). Genetic ancestry was estimated from PC analysis of genome-wide genotype data. Consistent with the expected composition of Mexican mestizo populations, the sample showed a mean Native American ancestry of 60.6% (SD = 7.1%, range 35-75%) and European ancestry of 39.4%, with minimal between-group variation by T2D status (T2D: 60.5% vs no T2D: 61.0%, *P* = ns; Fig. S2) (19).

The majority of participants (65.5%) were classified as having higher Native American ancestry (55-65%), while only 1.0% had predominantly European ancestry (<45% Native American). PC1 was strongly inversely correlated with Native American ancestry (*r* ≈ −0.99), validating its use as an ancestry covariate in regression models (Fig. S2) (19). Native American ancestry and PC1/PC2 were used in parallel as sensitivity analyses for population stratification correction (Tables 5 and 6, Fig. S2) (19).

### Sensitivity analysis: ancestry adjustment

Results were robust to the choice of ancestry covariate. Across all 3 FibroScan outcomes (CAP, steatosis grade, and FLI), substituting Native American ancestry percentage with PC1 and PC2 produced nearly identical coefficient estimates, SEs, and significance levels (Table 7, Fig. 2). The VAT × G/G interaction on steatosis grade remained significant in both covariates (Native American %: *P* = .021; PC1/PC2: *P* = .016), and the direction and magnitude of all estimates were consistent. PC1 was independently associated with lower CAP (*β* = −122.4, *P* = .030) and lower steatosis grade (*β* = −2.41, *P* = .048), confirming that greater European ancestry (reflected by higher PC1) is associated with lower hepatic steatosis burden, consistent with published ethnic differences in MASLD susceptibility. These findings confirm the *SLC16A11* rs13342232 × VAT interaction on hepatic steatosis.

**Figure 2:**
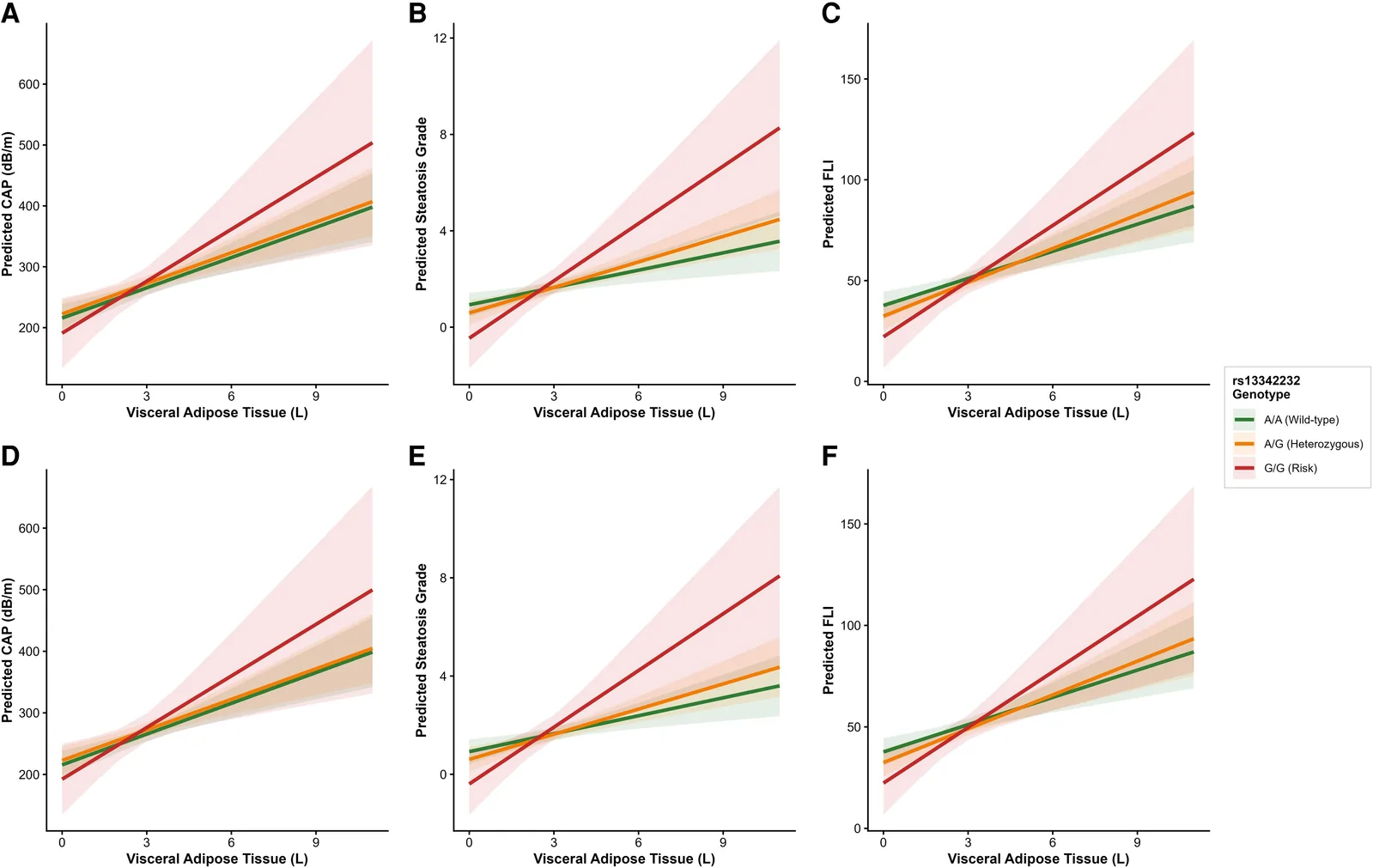
Sensitivity analysis of the interaction between visceral adipose tissue (VAT) and the *SLC16A11* rs13342232 genotype after alternative adjustment for genetic ancestry. Sensitivity analysis of the interaction between VAT and rs13342232 genotype on hepatic steatosis, comparing 2 methods of ancestry adjustment. (A-C) Models adjusted for the first 2 genetic principal components (PC1 and PC2). (D-F) Models adjusted for the proportion of Native American ancestry. Outcomes are controlled attenuation parameter (CAP, dB/m; A and D), steatosis grade (B and E) and fatty liver index (FLI; C and F). All models were also adjusted for age, sex, and type 2 diabetes; FLI models were also adjusted for body mass index. Lines show predicted values by genotype (A/A, reference; A/G; G/G), and shaded areas show 95% CIs. *n* = 301.

**Table 7:** Sensitivity analysis: VAT × *SLC16A11* rs13342232 interaction on CAP, steatosis grade, and FLI adjusted for genetic ancestry (PC1 and PC2)

	VM-CAP-PC (*n* = 301)	VM-Steatosis-PC (*n* = 301)	VM-FLI-PC (*n* = 301)
	*β* (95% CI)	*β* (95% CI)	*β* (95% CI)
Main effects			
VAT, L	16.567 (9.714, 23.419)***	.240 (0.091, 0.388)**	4.479 (2.324, 6.633)***
A/G (ref.: A/A)	6.674 (−24.442, 37.790)	−.340 (−1.013, 0.332)	−5.286 (−13.475, 2.904)
G/G (ref.: A/A)	−24.776 (−85.628, 36.077)	−1.386 (−2.703, −0.070)*	−15.521 (−31.539, 0.498)****
VAT × genotype interaction			
VAT × A/G	.213 (−8.757, 9.183)	.114 (−0.081, 0.308)	1.108 (−1.255, 3.471)
VAT × G/G	11.875 (−8.758, 32.508)	.554 (0.108, 1.000)*	4.722 (−0.713, 10.157)****
Ancestry			
PC1	−122.380 (−232.650, −12.110)*	−2.414 (−4.798, −0.029)*	−21.626 (−50.688, 7.435)
PC2	−51.890 (−195.830, 92.052)	−2.441 (−5.550, 0.668)	−5.975 (−43.860, 31.911)
Covariates			
Age, years	.530 (0.019, 1.040)*	.013 (0.002, 0.024)*	.034 (−0.100, 0.169)
Sex (male)	−13.828 (−30.234, 2.579)****	−.255 (−0.610, 0.099)	6.370 (1.756, 10.985)**
BMI, kg/m^2^	—	—	3.838 (3.347, 4.327)***
T2D	12.791 (−8.164, 33.746)	−.609 (−1.063, −0.156)**	−.690 (−6.208, 4.829)
Model fit			
*R*^2^	.21	.176	.741
Adjusted *R*^2^	.183	.147	.731
*n*	301	301	301
*F*-statistic (*P*-value)	7.70 (<.001)	6.18 (<.001)	75.02 (<.001)

Regression coefficients (*β*) represent unstandardized estimates with 95% CI (*β* ± 1.96 × SE). Reference category for rs13342232 was A/A (wild type). PC1 and PC2 were derived from genome-wide SNP data and included as covariates to account for genetic ancestry (Caucasian and Amerindian ancestry axes, respectively). Dashes (—) indicate variables not included in model.

Abbreviations: BMI, body mass index; CAP, controlled attenuation parameter; FLI, fatty liver index; PC, principal component; SNP, single-nucleotide polymorphism; T2D, type 2 diabetes; VAT, visceral adipose tissue.

*P < .05, **P < .01, ***P < .001, ****P < .10 (trend).

## Discussion

This study demonstrates that the *SLC16A11* rs13342232 G/G genotype significantly amplifies the effect of VAT on hepatic fat accumulation as measured by the FLI and FibroScan-derived steatosis grade in a Mexican mestizo population.

Our results extend prior knowledge of the *SLC16A11* haplotype and are consistent with a Chilean study, which reported associations of *SLC16A11* variants with increased BMI, insulin levels, and waist circumference in individuals without T2D (20). Notably, these results were observed in a population with a shared ancestral background, further supporting the role of *SLC16A11* in metabolic susceptibility. In addition, a study in Japanese individuals with T2D showed that the *SLC16A11* haplotype influences lipid metabolism, with carriers exhibiting higher total and low-density lipoprotein cholesterol levels compared with noncarriers; however, no evidence was found for an effect on liver function (21). These findings suggest that while the metabolic impact of *SLC16A11* is reproducible across diverse populations, its contribution to hepatic fat accumulation may be particularly evident in admixed groups with a high prevalence of visceral adiposity, as demonstrated in our study.

The biological mechanism underlying these associations remains to be fully elucidated. *SLC16A11* encodes a proton-linked monocarboxylate transporter expressed in the liver (2), and risk variants have been shown to reduce protein expression and alter intracellular lipid metabolism, potentially contributing to hepatic insulin resistance (4). A mechanism previously linked to an increased risk of T2D (2). In addition, impaired lipid handling may favor ectopic fat deposition, promoting visceral adiposity and increasing susceptibility to MASLD in genetically predisposed individuals. In this context, VAT may act as an amplifying factor, increasing the flux of free fatty acids to the liver and exacerbating fat accumulation in genetically susceptible individuals. These observations align with the broader concept of gene–environment interactions, where genetic variants predispose to disease only under specific metabolic conditions, such as excess visceral adiposity (5, 22, 23).

Regarding the T2D vs non-T2D groups, stratified analyses showed that although the T2D group presented a higher metabolic burden, including greater visceral adiposity, higher FLI, and higher CAP and liver stiffness values, the independent association between VAT and FLI was more pronounced in participants without diabetes. The G/G × VAT interaction on FLI was ∼4 times larger in magnitude in non-T2D participants (*β* = 7.51) than in T2D participants (*β* = 2.00, nonsignificant), and the interaction was replicated using FibroScan steatosis grade specifically in the non-T2D stratum (*β* = .502, *P* = .044). This gradient suggests that the susceptibility conferred by the G/G genotype is most clearly expressed in early stages of metabolic dysfunction, before the onset of diabetes, when visceral adiposity remains the dominant driver of hepatic lipid accumulation. The absence of a significant G/G × VAT interaction in T2D participants likely reflects that chronic hyperglycemia, insulin resistance, and the metabolic dysregulation associated with established diabetes independently overwhelm hepatic lipid handling capacity, reducing the marginal contribution of genotype. In this context, SLC16A11 may represent a gene that “primes” the liver for fat accumulation in response to visceral adiposity, with its effect becoming diluted once diabetes-related mechanisms dominate. The dominant model (A/A vs A/G + G/G) was null in the non-T2D stratum (*P* = .372), confirming a recessive inheritance pattern: 1 copy of the G allele is insufficient to significantly modify the VAT–steatosis relationship, and 2 copies are required. The negative and significant A/G × VAT interaction observed in the T2D stratum (*β* = −1.634, *P* = .046) is intriguing and may reflect a heterosis-like effect in the diabetic metabolic context, where heterozygotes exhibit attenuated VAT sensitivity relative to both homozygous classes. As observed in the diagnosed T2D group, in the FibroScan analysis, the inverse association between T2D and degree of steatosis may be due to unmeasured factors such as pharmacological treatment or disease duration, as reported in other studies.

In this study, the association between the *SLC16A11* variant and hepatic steatosis was assessed using the FLI. Fatty liver index integrates BMI, waist circumference, triglycerides, and GGT (16), variables that are strongly influenced by visceral adiposity and insulin resistance, 2 mechanisms directly linked to *SLC16A11* biology (4). In contrast, other commonly used noninvasive indices of hepatic steatosis rely more heavily on liver enzyme levels (16) or fasting glucose parameters (24, 25), which may not fully capture the combined anthropometric and metabolic alterations associated with this locus (5). The selectivity of the FLI signal thus provides internal biological validation of the specificity of the interaction. A potential concern is that BMI enters both FLI and the regression model as an independent covariate, raising the possibility of multicollinearity. However, GVIF analyses confirmed that all covariates, including BMI, had adjusted GVIF values well below the accepted threshold, indicating that the simultaneous presence of BMI in the index and as a covariate did not inflate SEs or distort effect estimates. Regarding the 5 *SLC16A11* haplotype variants, 3 of them (rs75493593, rs75418188, and rs117767867) produced virtually identical interaction estimates and *P*-values across all outcomes, consistent with high linkage disequilibrium within the risk haplotype. This expected redundancy supports reporting them as a single tagging unit. In the T2D-enriched cohort, these 3 variants showed a nonadditive (overdominant) interaction pattern with VAT on FLI: the T/T homozygote amplified the VAT–steatosis relationship (*β* ≈ +4.1), while the T/G heterozygote attenuated it (*β* ≈ −1.9), with both directions significant. This opposing dose–response pattern, where the heterozygote is not intermediate between the 2 homozygotes, is a hallmark of dominance deviation rather than additive allelic effects. Future studies should confirm pairwise *r*^2^ values in this population, and analyses of rs13342232 and rs13342692 should account for their complementary allele coding, as their interaction estimates appear as mirror reflections of each other, consistent with representation of the same locus on opposite strands.

Among the strengths of our analyses are the thorough genetic, biomedical, and anthropometric characterization of the study population, which provided adequate statistical power to examine gene-anthropometric interactions. The validity of the SNP × VAT interaction is further reinforced by 2 independent lines of converging evidence. First, a permutation-based dummy gene analysis—in which genotype labels were randomly reshuffled while preserving the original allele frequencies—yielded no significant interaction with VAT for any outcome (all interaction *P* > .20). Because model complexity, sample size, and covariate structure were identical to the real models, this result demonstrates that the significant interactions observed with the true genotypes reflect a biologically specific signal rather than a statistical artifact. Second, the statistically significant interaction between the rs13342232 G/G genotype and VAT was independently replicated in the FibroScan subsample using steatosis grade as the outcome (*β* = .526, 95% CI 0.080-0.972; *P* = .021), a biologically distinct instrument from FLI that does not share any mathematical components with it. This cross-modal replication across a nonoverlapping measurement platform substantially strengthens the robustness and external validity of the primary finding. Although transient elastography (FibroScan) is not considered the gold standard and has limited sensitivity for detecting mild steatosis, it is a well-validated noninvasive technique widely used in clinical and epidemiological research (26), further strengthening the reliability and external validity of our results. Adjustment for genetic ancestry (PC1/PC2) further minimizes the likelihood that population stratification explains the observed associations, which is particularly relevant in admixed populations such as Mexicans.

This study has certain limitations. First, we were unable to estimate other liver-related indices, such as fibrosis-4 (FIB-4) or liver fat score, as key variables, including platelet count and albumin levels (27), were not available. Second, the cross-sectional design precludes causal inference, and the validation sample, although consistent, was relatively small and may not fully capture the variability in genetic and metabolic profiles. Third, we did not assess diet, physical activity, or other environmental exposures. Additionally, detailed information about pharmacological treatment and duration of diabetes in participants with T2D was not available; these factors could further modify the observed associations. Furthermore, genetic ancestry was estimated using PCs derived from genome-wide genotype data. However, more precise ancestry estimation would require model-based admixture analyses (eg, ADMIXTURE) using well-characterized reference populations or validated ancestry-informative marker panels. The approach used assumes a simplified 2-way admixture model and does not explicitly quantify African ancestry contributions. Future longitudinal studies are needed to confirm whether *SLC16A11* variants and their interactions with visceral adiposity predict incident MASLD or its progression to advanced liver disease in individuals with T2D. Finally, participants without T2D were significantly younger than those with T2D (median 45 vs 60 years). This difference is consistent with the natural history of metabolic disease, wherein progressive visceral fat accumulation, insulin resistance, and systemic inflammation converge over time to drive the transition toward overt T2D and its associated cardiometabolic burden. Notably, the primary objective of this study was to characterize the extent to which the SLC16A11 genotype modifies the relationship between visceral adiposity and hepatic fat accumulation, a question for which age is a confounder to be controlled rather than an exposure of interest. Age was therefore included as a covariate in all multivariable models, ensuring that the gene–environment interaction estimates were not attributable to age-related differences in VAT or hepatic steatosis between strata.

### Conclusions

These findings advance our understanding of genotype-environment interactions in MASLD risk in T2D, supporting a model in which visceral adiposity acts as an amplifying trigger for hepatic fat deposition specifically in individuals carrying 2 copies of the *SLC16A11* risk allele.

## Data Availability

The datasets generated and/or analyzed during the current study are not publicly available due to participant privacy and institutional restrictions but are available from the corresponding author on reasonable request.

## Abbreviations

ALT: alanine aminotransferase
BMI: body mass index
CAP: controlled attenuation parameter
FFM: fat-free mass
FLI: fatty liver index
GGT: γ-glutamyl transferase
GVIFs: generalized variance inflation factors
INCMNSZ: Instituto Nacional de Ciencias Médicas y Nutrición Salvador Zubirán
MASLD: metabolic dysfunction–associated steatotic liver disease
PC: principal component
SNVs: single-nucleotide variants
T2D: type 2 diabetes
UIEM: Unidad de Investigación de Enfermedades Metabólicas
VAT: visceral adiposity tissue
